# Editorial: Advances on the Gametocyte Biology, Host Immunity and Vector Stages to Interrupt the Transmission of Malaria

**DOI:** 10.3389/fcimb.2022.918489

**Published:** 2022-05-20

**Authors:** Jo-Anne Chan, Priscilla Ngotho, Linda Eva Amoah, Lauriane Sollelis, Linda Reiling

**Affiliations:** ^1^Burnet Institute, Melbourne, VIC, Australia; ^2^Department of Medicine, Melbourne University, Parkville, VIC, Australia; ^3^Department of Immunology, Central Clinical School, Monash University, Melbourne, VIC, Australia; ^4^Wellcome Center for Integrative Parasitology, Institute of Infection and Immunity University of Glasgow, Glasgow, United Kingdom; ^5^Department of Immunology, Noguchi Memorial Institute for Medical Research, University of Ghana, Accra, Ghana; ^6^Wellcome Center for Integrative Parasitology, Institute of Infection and Immunity University of Glasgow, Glasgow, United Kingdom

**Keywords:** malaria vaccine, gametocytes, sexual differentiation, transmission blocking interventions, malaria elimination, malaria transmission

Stringent malaria control efforts have reduced the global malaria burden in the past decade. However, the decrease in case numbers has plateaued and increasing drug and insecticide resistance has made the development of new interventions a top priority. Further, this decrease has been unravelled by the on-going COVID-19 pandemic and the associated disruptions in access to global malaria interventions (WHO, 2021). In 2020, there were an estimated 241 million cases and 627,000 malaria deaths, an increase of over 14 million cases and 69,000 deaths compared to 2019 (WHO, 2021).

While much of the previous malaria control efforts have been focused on the pre-erythrocytic and blood-stages of malaria, specifically inhibiting the transmission of parasites from the human host to the mosquito vector is now recognized as a key step for elimination (MVF, 2013). Targeting the transmission stages of the parasite will confer protection on a population level by inhibiting the onward transmission from an infected individual to the next.

The transmission stages of malaria are complex and offer multiple opportunities for intervention. Various environmental and genetic signals trigger the switch from asexually replicating forms within the human host to sexual gametocytes. In the human host, gametocytes, still residing within red blood cells, sequester and develop in the extra-vasculature of hematopoietic niches, such as the bone marrow and spleen. At maturation, male and female gametocytes are released back into the peripheral circulation and can be taken up by mosquitoes during blood feeds to complete transmission from human to mosquito. In the mosquito midgut, male and female gametes emerge to undergo sexual replication through fertilisation, resulting in the formation of zygotes. These develop into actively moving ookinetes that move through the midgut epithelium to develop into oocysts in the surface of the midgut. Upon maturation, oocytes burst to release sporozoites into the body cavity which then migrate to the salivary glands to start a new cycle of infection upon the next blood meal. Antigens that are expressed during this stage of the parasite life cycle, specifically on the surface of gametocytes and gametes, represent attractive targets of antibodies, which can contribute to the development of effective vaccines (Stone et al., 2018).

The reviews and original research articles in this Research Topic feature the recent progress in this understudied area and highlight important knowledge gaps that need addressing, in order to develop new transmission blocking interventions. Articles in the first section describe the natural acquisition of antibodies to antigen targets on the surface of gametocytes in endemic populations. Muthui et al. demonstrated the acquisition of human antibodies to gametocyte surface antigens, including the lead vaccine candidate Pfs230 using two clinical cohorts from an endemic region in Kenya. Further, they identified a panel of new gametocyte antigens with the potential to be further evaluated as transmission-blocking vaccine candidates. Broni et al. examined the levels and avidity of naturally induced antibody responses against vaccine candidates Pfs48/45 and Pfs230, from the end of a peak malaria season to the start of the subsequent season. O’Flaherty et al. examined associations between antibody levels to Pfs230 and Pfs48/45 and concurrent gametocytemia across different transmission settings using human samples from a multi-national clinical study. They showed that Pfs230 and Pfs48/45 are targets of natural immunity that are associated with gametocyte densities across multiple endemic settings.

Takashima et al. reviewed the progress and challenges in identifying new and effective transmission-blocking vaccine candidates. To accelerate the process of antigen discovery for transmission-blocking vaccines, they proposed the use of genome-wide approaches to identify key target antigens. These will be subsequently expressed using a recombinant system that produces correctly folded proteins to evaluate for transmission-blocking activity. Another review article by Yu et al. highlights the potential of biological control strategies that directly kill the mosquito vector as an effort to interrupt malaria transmission. They further discuss the ongoing infectivity of patients after clearance of blood stage parasitemia as gametocytes are not effectively targeted by the current gold-standard ACT treatment. The only currently licensed drug that kills gametocytes is Primaquine, but wide usage is prohibited by safety issues in G6PD deficient patients. Atovaquone/Proguanil have been shown to have transmission blocking activity by targeting mosquito stages of the parasites, however a short drug half-life is problematic. These are areas where further research is needed and offers potential for further targeted intervention.

The next section of articles comprises original research and review articles on the commitment to sexual development and transmission dynamics. *Plasmodium* parasites are able to adjust commitment to gametocyte conversion and investment in transmission in areas of changing environments. Importantly, investment into gametocytes for both *P. falciparum* and *P. vivax* has been shown to be higher in areas of lower transmission (Rono et al., 2018, Koepfli et al., 2021), whereas seasonal changes appear to have an opposite effect with higher transmission in the wet season (reviewed in Oduma and Koepfli ). This adaptation to changes in transmission, and in particular differences between Pf and Pv transmission biology needs to be addressed when designing novel transmission blocking interventions. Methodologies to assess gametocyte conversion rates, transmission potential and gametocyte infectivity are summarized in Oduma and Koepfli l., while Ford et al. presented novel biomarkers of gametocyte presence. Thommen et al. addressed the important question of whether drug induced stress responses can lead to an increased commitment to sexual development. Usui and Willimson. provide a comprehensive review and discuss transmission dynamics and identify knowledge gaps. Timinao et al. investigated the infectivity of different human to malaria reservoirs to estimate their contribution to the transmission of malaria. They recruited symptomatic patients from an endemic region in Papua New Guinea and showed through direct membrane feeding assays that the majority of gametocyte-positive individuals were able to infect *Anopheles* mosquitoes. Further, they showed that acute *P. vivax* infections led to more frequent mosquito infections as their gametocytes developed much quicker than those of *P. falciparum*.

Together, the collection of articles in this Research Topic emphasizes the importance of further research into transmission-blocking approaches that is key for achieving sustained malaria elimination.

## Author Contributions

J-AC and LR contributed equally to writing the manuscript. PN edited sections of the manuscript. All authors contributed to manuscript revision, read, and approved the submitted version.

## Conflict of Interest

The authors declare that the research was conducted in the absence of any commercial or financial relationships that could be construed as a potential conflict of interest.

## Publisher’s Note

All claims expressed in this article are solely those of the authors and do not necessarily represent those of their affiliated organizations, or those of the publisher, the editors and the reviewers. Any product that may be evaluated in this article, or claim that may be made by its manufacturer, is not guaranteed or endorsed by the publisher.

## References

[B1] Koepfli C. Nguitragool W. de Almeida A. C. G. Kuehn A. Waltmann A. Kattenberg E.. (2021). Identification of the Asymptomatic Plasmodium Falciparum and Plasmodium Vivax Gametocyte Reservoir Under Different Transmission Intensities. PloS Neglected Trop. Dis. 15 (8), e0009672. doi: 10.1371/journal.pntd.0009672 PMC8428688 34449764

[B2] Malaria Vaccine Funders Group. (2013). Malaria Vaccine Technology Roadmap. Available at: https:www.malariavaccine.orgmalaria-and-vaccinesmalaria-vaccine-roadmap

[B3] Rono M. K. Nyonda M. A. Simam J. J. Ngoi J. M. Mok S. Kortok M. M.. (2018). Adaptation of Plasmodium Falciparum to Its Transmission Environment. Nat. Ecol. Evol. 2 (2), 377–387. doi: 10.1038/s41559-017-0419-9 29255304 PMC7116667

[B4] Stone W. J. R. Campo J. J. Ouedraogo A. L. Meerstein-Kessel L. Morlais I. Da D.. (2018). Unravelling the Immune Signature of Plasmodium Falciparum Transmission-Reducing Immunity. Nat. Commun. 9 (1), 558. doi: 10.1038/s41467-017-02646-2 29422648 PMC5805765

[B5] World Health Organisation. World Malaria Report. (2021).

